# Psychiatric Disorders and lncRNAs: A Synaptic Match

**DOI:** 10.3390/ijms21093030

**Published:** 2020-04-25

**Authors:** Francesco Rusconi, Elena Battaglioli, Marco Venturin

**Affiliations:** Dipartimento di Biotecnologie Mediche e Medicina Traslazionale, Università degli Studi di Milano, Via Fratelli Cervi 93, 20090 Segrate, Italy; elena.battaglioli@unimi.it

**Keywords:** lncRNAs, neuropsychiatric disorders, epigenetics, synaptic function, evolution, environmental adaptation, homeostasis

## Abstract

Psychiatric disorders represent a heterogeneous class of multifactorial mental diseases whose origin entails a pathogenic integration of genetic and environmental influences. Incidence of these pathologies is dangerously high, as more than 20% of the Western population is affected. Despite the diverse origins of specific molecular dysfunctions, these pathologies entail disruption of fine synaptic regulation, which is fundamental to behavioral adaptation to the environment. The synapses, as functional units of cognition, represent major evolutionary targets. Consistently, fine synaptic tuning occurs at several levels, involving a novel class of molecular regulators known as long non-coding RNAs (lncRNAs). Non-coding RNAs operate mainly in mammals as epigenetic modifiers and enhancers of proteome diversity. The prominent evolutionary expansion of the gene number of lncRNAs in mammals, particularly in primates and humans, and their preferential neuronal expression does represent a driving force that enhanced the layering of synaptic control mechanisms. In the last few years, remarkable alterations of the expression of lncRNAs have been reported in psychiatric conditions such as schizophrenia, autism, and depression, suggesting unprecedented mechanistic insights into disruption of fine synaptic tuning underlying severe behavioral manifestations of psychosis. In this review, we integrate literature data from rodent pathological models and human evidence that proposes the biology of lncRNAs as a promising field of neuropsychiatric investigation.

## 1. Neuropsychiatric Disorders: One for All and All for One

Psychiatric illnesses represent a heterogeneous class of diseases. The most common disorders include major and bipolar depression (MDD and BD), many forms of persistent anxiety (including obsessive compulsive disorder (OCD), post-traumatic stress syndrome (PTSD), and panic disorders), addictive disorders, schizophrenia (SCZ), autism spectrum disorders (ASD), intellectual disability (ID), and attention-deficit/hyperactivity disorder (ADHD). Symptoms include psychosis, delusions, motivation loss, repetitive behaviors, uncontrolled anxiety, cognitive issues, and dissociation, all of which translate into severely affected ability to function and live a productive and satisfying life. Their incidence has grown to worrying percentages, cumulatively reaching 20% of the population in Western countries. Psychiatric disorders often originate from pathological associations between a certain genotype, involving susceptibility under the form of multiple detrimental hypomorphic gene mutations or vulnerability-related Single Nucleotide Polymorphisms (SNPs) and negative environmental contingencies, grouped together as environmental stressors. The genetic component of neuropsychiatric disorders emerged from family studies, clearly showing hereditability aspects [1,2]. However, as no clear examples of Mendelian inheritance in mental illness have been provided [1,3], it is widely accepted that genetic substrates involve multiple alleles, each contributing with a small effect, thus defining mental diseases as complex genetic disorders [1,3]. Notably, environmental stressors thoroughly contribute to these pathologies. This is well documented by the low phenotypic concordance of neuropsychiatric traits among monozygotic twins, which for SCZ only reaches a rate of about 50% [4,5]. Accordingly, many studies describe environmental stress as a relevant risk or triggering factor for mental diseases [2,6,7,8].

Mental illnesses feature several shared behavioral symptoms, but they also display disease-peculiar traits that are used to refine their classification. For instance anxiety, a highly common morbidity experienced by depressed, schizophrenic, and obsessive-compulsive patients, among others, represents a core behavioral alteration. Similarly, paranoia, cognitive issues, and delusion can be manifested within a smaller subset of diseases, such as BD and SCZ [9]. Another layer of neuropsychiatric disorder complexity is represented by alternation of opposite polarity symptoms, as in BD patients, such as manic or depressive episodes [10].

With this said, as elusive dividing lines characterize different neuropsychiatric disorders in terms of symptoms association, univocal diagnoses are often difficult to draw [1,3]. Non-trivial behavioral categorization of mental illness is coupled to other recurrent features displayed by neuropsychiatric patients, including alternation of symptomatic manifestations with moments of relative normality and sociality, which again often precipitate following real or perceived negative context or experiences. Moreover, classification of mental disorders cannot account on objective criteria, as no molecular and morphostructural correlates have yet been listed as diagnostic biomarkers. Neuropsychiatric disorders are, therefore, recognized in accordance with International Classification of Disease (ICD) and Diagnostic and Statistical Manual (DSM) guidelines by arbitrary (though accurate) symptoms and signs by clinicians. This evidence poses the question of how (and if) to distinguish mental disorders from one another [9]. A lack of objective diagnostic criteria not only limits mental illness classifications, but also suggests that a deeper knowledge of molecular and structural endophenotypes (specific, measurable, and quantifiable biological hallmarks) would represent a fundamental step to reliable diagnosis and a step forward in disease pathogenesis and future therapies [9].

Interestingly, within heterogeneous mental illness symptomatology, we suggest a unifying origin under the form of *abnormal environmental interaction*. Within this domain, it is indeed possible to identify two specific features: (i) psychiatric patients display aberrant perception of contingencies and experiences, pathologically arising from an inability to make valid predictions [11,12]; and (ii) psychiatric patients show severe impairment of environmental adaptability. For example, generalized anxiety patients are frightened and engage in avoidance-like behaviors following neutral or unthreatening conditions. In line with this, hallucinations in schizophrenic patients could be seen as an extreme exacerbation of the described misinterpretation [11]. Regarding the second aspect, impairment of environmental adaptability, patients suffer from a lack of psychological equilibrium, making them particularly vulnerable to and modification of their habits. Repetitive behaviors, along with more complex obsessive manifestations, both result from a stubborn refusal to deviate from routine, regardless of the environment.

From a biological point of view, our interaction with the environment is mediated at the cellular level by the synapses. Deciphering the nature of impaired synaptic function, i.e., synaptic endophenotypes [9], represents a crucial way to increase our knowledge about mental illness pathophysiology.

## 2. Neuropsychiatric Disorders: Genes and Synaptic Endophenotypes—A Gap to Fill

From the genetic point of view, remarkable convergence has recently been observed within multiple psychiatric disorders, indicating that similar molecular pathways are involved in different forms of mental illness, also providing a functional perspective on the above-mentioned overlapping symptoms [13,14,15,16]. One of these studies took advantage of exome sequencing analyses, which were able to unveil how the same genes and pathways are preferential targets of de novo mutations within patients of three different disorders—ASD, SCZ, and ID [13]. Gene ontology fell into the categories of neuron differentiation, neurogenesis, chromatin modification, and synapse functional modulation. Another work based on genome-wide association studies (GWAS) provided empirical evidence of shared genetic etiology for five psychiatric disorders, namely BP, MDD, ADHD, SCZ, and ASD, providing further evidence of common pathophysiologic mechanisms for these related disorders falling into the same above-mentioned functional categories [15]. Interestingly, synaptic genes and chromatin regulators are emerging as pivotal markers in the pathogenesis of mental illness [13,17]. A number of synaptic genes are indeed targeted by de novo mutations in this set of pathologies, from receptors to ion channels and scaffolding proteins, including sodium channel *SCN2A*, the *N*-methyl-d-aspartate receptor (NMDAR) subunit *GRIN2B*, and *SHANK* genes [16,17]. Additionally, adhesion molecules, such as neurexin (NRXN) and neuroligin (NLGN), have been reported to play important pathogenic roles in mental disorders [18]. Interestingly, the family of epigenetic modifiers involved in transcriptional regulation via chromatin remodeling of synaptic targets is also important in neuropsychiatric disorders. In this regard, histone demethylases seem to occupy prominent positions; for example, KDM5B and KDM6B [16,17] together with KDM1A [19] seem to play a part in autism-related symptoms, and notably represent causative genes for different forms of intellectual disability [20]. It is also interesting to report the implications of KDM1A in neuropsychiatric-relevant behaviors, such as anxiety, in addition to learning and memory formation [21,22,23,24].

Interestingly, many recent studies based on RNA sequence analyses have highlighted transcriptional deregulation of long non-coding RNAs (lncRNAs) in the post-mortem brains of psychiatric patients. This evidence is consistent with recent discoveries, showing that many mutations linked to neuropsychiatric disorders fall into non-coding regions of the genome. In this context, it is particularly compelling to underline the growing importance of a new class of non-coding genes, lncRNAs, whose role in synaptic function (see below) encourages investigation of their role in neuropsychiatric disorders.

Indeed, proper functioning and regulation of synaptic transmission, largely contributed by the mentioned classes of coding and possibly non-coding genes, is fundamental to adaptive neurophysiology, which is why alteration of fine synaptic tuning as a substrate of mental illness is a tempting yet promising hypothesis. Interestingly, fine synaptic tuning not only accounts for cell-autonomous mechanisms, but also complex circuitry and neuroendocrine-system-related modulation. In the following paragraphs, we provide a brief description of three known endophenotypes with established roles in neuropsychiatric disorders, whose potential lncRNA-dependent regulation could reveal remarkable cues for pathomechanisms, and hence treatment of mental illness.

### 2.1. HPA Axis Dysfunction

Established theories link synaptic maladaptive plasticity to neuroendocrine dysfunctions, and in particular to aberrant glucocorticoid metabolism and signaling related to hypothalamic–pituitary–adrenal (HPA) axis deregulation [25]. As mentioned, stress, particularly when chronically reiterated, leads to excessive HPA axis stimulation, entailing desensitization of the negative feedback loop, which is instrumental to stress response termination [26]. Noticeably, such aberrant glucocorticoid signal transduction causes profound issues in the glutamatergic synapse, having serious detrimental effects on both axon terminations in terms of aberrant homeostasis of glutamate release, and at the level of dendritic spines in terms of structural modifications, shrinkage, and decreased density [27,28]. As a matter of fact, stress-induced glucocorticoid disruption participates in the generation of a psychiatric-like impoverished neurostructural landscape in important brain areas and is widely recognized to play a fundamental role in mental illness pathogenesis, such as the medial prefrontal cortex, the amygdala, and the hippocampus [29,30].

### 2.2. The Endocannabinoid System Disruption

Another interesting synapse-related set of processes likely involved in the pathogenesis of neuropsychiatric disorders is represented by the endocannabinoid system (ECS) [31,32,33]. The ECS is involved in stress response termination as part of a homeostatic mechanism aimed at decreasing the amount of stress-elicited neurotransmitter release, in the context of bringing synapse physiology back to baseline, shutting down anxiety arousal in the meantime. Interestingly, ECS has been shown to be targeted by chronic stress. Within stress reiteration, indeed endocannabinoid 2-arachidonyl glycerol (2-AG), the prominent ligand of cannabinoid receptor 1 (CB1), is consistently synthesized in the post-synapse, causing CB1 desensitization in the pre-synapse and relative loss of control over neurotransmitter release [34]. This maladaptive pathway represents one of the gateways of emotional drift shared by most neuropsychiatric disorders. Additionally, 2-AG’s half-life, which is enhanced upon acute stress by a transcriptional mechanism aimed at decreasing its degrading enzymes MAGL and ABHD6, is not positively regulated when exposed to chronic stress [35].

### 2.3. Predictive Sensing Alteration

Brain activity features peculiar default oscillations (including gamma oscillations), whose synchronization with external stimuli, known as predictive sensing, optimizes environmental perception, response, and adaptation. Predictive sensing is referred to as a highly dynamic mechanism entraining default patterns of internal excitability fluctuations to external stimuli, thereby amplifying inputs related to these stimuli [11,12]. Interestingly, there is substantial agreement about gamma oscillations and predictive sensing disruption in SCZ [12,36], a process that requires fine synaptic tuning.

As mentioned, the available literature still cannot provide readers with a precise description of lncRNAs’ biological contribution to the three above-mentioned core endophenotypes of neuropsychiatric disorders. However, we collected evidence suggesting inherent involvement of lncRNAs in cellular neurobiology processes, whose emergent properties underlie prototypic neuropsychiatric endophenotypes. Therefore, to better understand the role of lncRNAs in neuropsychiatric disorders, we suggest the importance of actually studying their biological relevance from the perspective of aberrant functioning of HPA axis regulation, the endocannabinoid system, and the process of predictive sensing.

In the next sections we will (i) describe the biology of lncRNAs and then (ii) provide a perspective on how some of them, in particular GOMAFU, NEAT1, and MALAT1, could play important roles in synaptic tuning and mental illness.

## 3. Evolution and Biology of Long Non-Coding RNAs

The application of next-generation sequencing (NGS) technology to transcriptome analysis has radically changed the way we look at our genome, revealing that what was previously considered “junk DNA” due to a lack of coding potential is actually actively transcribed, giving rise to thousands of non-coding RNA transcripts with regulatory roles. It is estimated that more than 80% of the human genome is actively transcribed but only around 2%–3% is translated into proteins, meaning that the transcriptional output of our genome is almost monopolized by non-coding RNAs (ncRNAs) [37]. Long non-coding RNAs represent the most abundant class of ncRNAs in the human genome. The current GENCODE release (version 33) [38] contains 17,952 lncRNA genes, accounting for 48,438 transcripts, while the number of lncRNAs annotated by the NONCODE (v5.0) and LNCpidia (version 5.2) databases rises to 96,308 and 56,946, respectively [39,40]. Remarkably, while the number of protein-coding genes and corresponding sequences remained surprisingly constant during the evolution of eukaryotic organisms, the number of lncRNAs dramatically rose with increasing developmental and organ complexity (Table 1) [41,42]. Similarly, the set of “synaptic genes”, which mechanistically fulfill neuronal core biological functions, is largely conserved among the different rungs of the evolutionary ladder, even concerning very simple nervous systems, such as in C. elegans [43]. On the contrary, if we consider that Homo sapiens holds 8000 additional lncRNAs compared to Mus musculus and that almost half of them are brain specific [44], we can easily appreciate that among the same class of organisms (mammals), variability of lncRNAs correlates with increased complexity and brain connectomics [45]. A significant fraction of these newly emerged lncRNAs is involved in transcriptional and epigenetic control of gene expression, acting on nuclear organization and chromatin states, among other functions. Thus, it has been hypothesized that lncRNAs had a crucial role in creating the high complexity of the regulatory mechanisms and networks typical of the most evolved organisms. This is further supported by the observation that many lncRNAs have more specific expression patterns than coding genes, especially in the brain [44].

### 3.1. General Features of Long Non-Coding RNAs

LncRNAs are generally defined as non-coding RNA molecules longer than 200 nucleotides, a threshold that conventionally distinguishes them from small non-coding RNAs, the other group of ncRNAs, which includes tRNAs, rRNAs, snRNAs, and snoRNAs, as well as miRNAs, siRNAs, and piRNAs. LncRNAs share some similarities with mRNAs—they are usually transcribed by RNA polymerase II, capped with a 7-methylguanosine at the 5′ end, and spliced; most of them are also polyadenylated at the 3′ end and have their own promoter regions. Differing from protein-coding mRNAs, most lncRNAs lack an open reading frame (ORF), do not produce detectable peptides [46,47], and contain fewer exons [47,48].

LncRNAs display low primary sequence conservation compared to both protein-coding genes [49] and small ncRNAs [50], while they exhibit a high degree of conservation of their secondary structure [51]. Indeed, the functional role of specific lncRNAs can be preserved despite modest sequence conservation [50,52]. Several human and mouse lncRNAs have consistently been shown to phenotypically rescue the loss of function of zebrafish homologs, demonstrating functional conservation across species [53]. LncRNAs also have highly conserved splice junction sites [54]. Even more interestingly, the degree of conservation of their promoter regions is similar to that of coding genes [49]. These highly conserved promoters bind transcription factors and are subjected to chromatin remodeling events that correlate with their tissue-specific expression patterns [47,49]. RNA sequence experiments have shown that the expression of lncRNAs is lower and more tissue- and differentiation-stage-specific when compared to the expression of protein-coding genes, suggesting that they may have a role in fine modulation of cell fate and identity, thus providing a fundamental contribution to the formation and functioning of tissues and organs [55]. This is particularly true for the human brain, which expresses the largest number of lncRNAs [44].

### 3.2. Classification and Mechanisms of Action of Long Non-Coding RNAs

The classification of lncRNAs is not an easy task. Various methods to classify them have been proposed based on their different properties [56]. Typically, lncRNAs are grouped into different categories according to the relationship between their position and the location of the neighbouring protein-coding genes, even if peculiar classes of lncRNAs do not fit into this positional classification as they present a combination of mentioned features. Based on location, lncRNAs can be classified as: (1) Long intergenic non-coding RNAs (lincRNAs), defined as lncRNAs that do not overlap annotated coding genes. LincRNAs represent the largest and most significant group of lncRNAs, constituting approximately half the overall number of lncRNA [57]. (2) Sense overlapping lncRNAs, which are transcribed from the sense strand of a protein-coding gene. They may partially or totally overlap with the protein-coding genes and they can contain exons from protein-coding genes. In some cases, they are considered isoforms of the protein-coding transcript. (3) Antisense overlapping lncRNAs, also known as natural antisense transcripts (NATs), which are transcribed from the opposite DNA strand to a protein-coding transcript with variable degrees of overlap of coding exons. Interestingly, transcriptome analyses suggest that at least 70% of coding genes have an antisense counterpart [58]. (4) Bidirectional ncRNAs (bincRNAs), which have a head-to-head orientation with a protein-coding gene and do not overlap or only partially overlap with its 5′ region. A bincRNA transcript normally exhibits an expression pattern similar to that of its host gene, suggesting that they may be subjected to common regulatory mechanisms [59]. (5) Intronic ncRNAs, which are transcribed from the introns of annotated protein-coding genes in either a sense or antisense direction. MacroRNAs are very long RNAs that encompass huge genomic distances and numerous genes, or even the whole chromosome [60]. Circular RNAs (circRNAs) are a class of recently discovered regulatory RNAs produced by back-splicing circularization of pre-mRNA exons [61]. Circular intronic RNAs (ciRNAs) are created from excised introns, presenting specific sequences that avoid debranching of the lariat introns. Two other classes of lncRNAs, the promoter upstream transcripts (PROMPTs) and enhancer-associated RNAs (eRNAs), are transcribed from gene promoters or enhancers, respectively [62].

A growing number of studies have shown that lncRNAs have a wide spectrum of functions and are involved in several biological processes, notably differentiation and development [63,64]. They are able to control gene expression at multiple levels—epigenetic, transcriptional, and post-transcriptional—and participate in many other important cellular functions, including X-chromosome silencing, genomic imprinting, and chromatin modification [63,65,66,67]. This remarkable versatility of lncRNAs is due to their dual ability to recognize and bind specific DNA or RNA sequences through base-pairing interactions and to fold into secondary or higher-order structures that mediate their interaction with proteins. In this way, lncRNAs behave as flexible and modular scaffolds for single proteins or protein complexes and can guide them to specific DNA or RNA targets. A detailed description of the diverse mechanisms of action and cellular functions of lncRNAs goes beyond the scope of this review article (see [68,69] for comprehensive review). LncRNAs can exert their functions in both the nucleus and the cytoplasm, and their different mechanisms include the modulation of the chromosome architecture, the control of interactions between distant genomic regions, interaction with chromatin-modifying complexes, conformation of nuclear domains, activation of transcriptional enhancers, interference with the transcriptional machinery, and the structural formation and maintenance of nuclear bodies, such as speckles and paraspeckles. Furthermore, lncRNAs can act at the post-transcriptional level as regulators of splicing, mRNA decay, protein translation, protein stability, or as decoys or sponges for miRNAs. This latter function inspired the so-called “competing endogenous RNA (ceRNA) hypothesis”, according to which coding and non-coding RNAs can crosstalk through their ability to compete for miRNA binding (with miRNA target sites viewed as the “letters” of a common language), thus reciprocally influencing their respective expression levels [70].

### 3.3. Long Non-Coding RNAs in the Central Nervous System: High Versatility and Specificity to the Service of High Complexity

The human brain, and particularly the cerebral cortex, is undoubtedly the most complex and sophisticated organ that ever evolved. The correct development of the brain implies a precise spatiotemporal regulation and a proper balance of pluripotent stem cell proliferation and differentiation in order to form a spatially rigorous yet dynamic circuitry. A huge number of neurons take part in these circuits, forming the appropriate synaptic connections. They are also capable of changing these synaptic connections and their relative strength over time in response to both internal signals and external sensory experiences, thus mediating the highest levels of CNS functions, such as cognition and behavior.

Significantly, the emergence of this complexity and plasticity was paralleled during evolution of the CNS by the appearance of thousands of new primate-specific or even human-specific lncRNAs. Indeed, it was calculated that about one-third of human lncRNAs are specific to the primate lineage [47], hundreds of which seem to be human-specific [71], in stark contrast to the majority of protein-coding genes expressed in the nervous system, which is highly conserved among mammals [72,73,74] (see Table 1). Remarkably, about 40% of the lncRNAs identified in the human genome (corresponding to 4000–20,000 lncRNA genes) are specifically expressed in the brain, a number which is strikingly large when considering that the human genome contains a total of about 20,000 protein-coding genes and around 2300 miRNAs [75], with only a subset of these being specific to the CNS. Additionally, lncRNAs expressed in the brain show greater spatiotemporal, cell-type, and subcellular expression pattern specificity than protein-coding genes [76,77,78], and their expression is dynamically regulated during development [79,80,81] in response to neuronal activity [82,83,84] and during aging [85]. Another interesting feature of brain-expressed lncRNAs is their preferential genomic position in the proximity of brain-specific coding genes that are important for transcriptional regulation during CNS development, often sharing with them a similar expression pattern, indicating that they probably exert an essential role in fine modulation of the levels of genes that drive neurogenesis [81,86].

The high regulatory versatility of lncRNAs, the high specificity of their expression profile, and the emergence of new lncRNAs during primate and human evolution probably made these transcripts crucial actors driving human-specific brain features by enhancing neuronal spatiotemporal gene regulation, a prerequisite for the brain’s capability to solve complex neurobiological tasks [87].

### 3.4. Role of Long Non-Coding RNAs in Neuronal Differentiation

Recent functional studies have revealed that a large number of lncRNAs play pivotal roles in all stages of neuronal differentiation, from pluripotent cells in the early embryo to the terminal cell types in the mature brain [88]. The underlying regulatory mechanisms are generally based on the modulation of these lncRNAs by canonical pluripotency transcription factors, such as OCT4, SOX2, and NANOG; in turn, lncRNAs exert their regulatory functions by directing transcription factors or chromatin remodeling complexes to lineage-specifying genes [89].

A number of studies using in vitro model systems such as embryonic stem cells (ESCs) or carried out in vivo in the embryonic mouse brain have demonstrated that several lncRNAs are involved in establishing pluripotency or driving neural lineage entry. For example, the lncRNA rhabdomyosarcoma-2-associated transcript (RMST), which is regulated by the transcriptional repressor RE1-Silencing Transcription factor (REST) and is restricted to the brain, was found to modulate the differentiation of neural cells in vitro. RMST acts by recruiting the neural transcription factor SOX2 to key neurogenic genes, such as *DLX1*, *ASCL1*, *HEY2*, and *SPS*. Accordingly, the loss of RMST can block the exit from the ESC state and the initiation of neural differentiation [90]. The lncRNA named TCL1 upstream neural differentiation-associated RNA (TUNAR, also known as TUNA) is also able to affect gene expression in neural cells by a similar mechanism. It can interact with NCL, PTBP1, and hnRNP-K RNA-binding proteins and direct them to the promoters of neural target genes in differentiating mouse ESCs. Knockdown of TUNA or each of these three interacting RNA-binding proteins is sufficient to inhibit neural differentiation [91]. Strikingly, the same functional role is also present in mice and zebrafish, indicating that the neural lineage commitment driven by lncRNAs is conserved across relatively distant vertebrates [88]. TUNA and RMST are examples of lncRNAs that can control cell-fate choice by directing transcription factors and chromatin-remodeling complexes to target loci, allowing the orchestration of complex gene expression programs by a single lncRNA. It has been proposed that this is the modus operandi of many lncRNAs during neural differentiation [92]. The lncRNA PNKY is expressed in the nucleus of dividing neural stem cells (NSCs) in developing mouse and human brains, where it acts as a regulator of NSC turnover by controlling the balance between self-renewal and neuronal differentiation in dividing NSCs. PNKY knockdown leads to an increase in neuronal differentiation and to the depletion of the NSC population in the embryotic mouse cortex. PNKY performs this function by regulating the alternative splicing process through the interaction with PTBP1, an alternative splicing regulator that represses the inclusion of neural exons in non-neural cells [93]. Another lncRNA with a well-established regulatory role in neural development is EVF2 (or DLX6-AS1). Genetic depletion in EVF2 causes an imbalance of the excitatory to inhibitory neurons in the postnatal hippocampus and dentate gyrus, owing to the failure of GABAergic interneuron specification, also leading to reduced synaptic inhibition [94]. EVF2 acts by recruiting the DLX transcription factor and methyl CpG-binding protein MECP2 to regulatory regions controlling the expression of interneuron lineage genes (including *DLX5*, *DLX6*, and *GAD1*) needed for GABAergic interneuron specification in the developing mouse forebrain [94,95]. The intergenic lncRNA linc-BRN1B controls the differentiation of delaminating neural progenitor cells in vivo. Deletion of the linc-BRN1B locus results in significant loss of upper cortical layers and of the expression levels of POU3F3, a transcription factor implicated in neuronal development. This reduction originates with loss of basal cortical progenitors, which subsequently drives precocious migration and differentiation of lower layer neurons [96]. GOMAFU (also known as MIAT, myocardial infarction associated transcript) is a lncRNA expressed in the nucleus of dividing NSCs and differentiating neurons. It modulates the alternative splicing of several neuronal genes, including the schizophrenia-associated genes *DISC1* and *ERBB4*, likely through the interaction with the splicing factors SF1, SRSF1, and QKI [84,97]. Depletion of GOMAFU in embryonic mice leads to increased amacrine cell and Muller glia differentiation [98], as well as an improperly regulated transition of actively dividing ventricular zone progenitor cells into differentiating neurons as they migrate outward into the cortical plate [81].

Altogether, these findings point to lncRNAs as key regulators of neural cell fate specification and stem cell turnover during brain development by conducting lineage-specific gene expression programs [88].

### 3.5. Role of Long Non-Coding RNAs in Neurite Elaboration and Synaptogenesis

LncRNAs are also implicated in neurite outgrowth and synaptogenesis, which are critical stages during brain development in order to establish the connections required for normal brain function [88]. An antisense lncRNA, BDNF-AS, which transcribes antisense to the *BDNF* growth factor gene, can control neurite elaboration during development through the regulation of local gene expression. Indeed, inhibition of BDNF-AS resulted in an increase in BDNF protein levels, which was associated with reduced EZH2 recruitment and altered chromatin state at the BDNF locus. The resulting BDNF overexpression then drove elevated neuronal outgrowth, differentiation, survival, and proliferation, both in vitro and in vivo [99]. The first lncRNA that was found to be involved in the regulation of synaptogenesis is BC1/BC200 (also named BCYRN1, brain cytoplasmic RNA 1). BC1/BC200 is expressed in the developing and adult CNS, where it is actively trafficked to dendrites [100]. There, it interacts with FMRP and the translational machinery, where it represses local translation in the synapse [101]. Through this mechanism, BC1/BC200 regulates spatially restricted synaptic turnover in vivo [102,103,104]. The lncRNA metastasis associated lung adenocarcinoma transcript 1 (MALAT1) is abundantly expressed in neurons and is enriched in nuclear speckles in a transcription-dependent manner. MALAT1 has been shown to recruit Ser/Arg (SR) family splicing proteins to transcription sites with the aim of controlling the expression of synaptogenesis-associated genes in cultured hippocampal neurons. Moreover, knockdown of MALAT1 in this system reduces synaptic density levels, while its overexpression increases synaptic density [105]. These data suggest that MALAT1 is able to regulate synaptogenesis by modulating synapse formation or maintenance genes via a mechanism involving splicing modulation via tethering splicing factors to nuclear speckles.

### 3.6. Role of Long Non-Coding RNAs in Neuronal and Synaptic Plasticity

LncRNAs are also involved in the regulatory mechanisms that govern synaptic plasticity and strength. Synaptic plasticity plays an important role in learning and memory processes. The learning process in embryonic and adult brains depends on normal development of a correct balance between excitatory glutamatergic and inhibitory GABAergic neurons in the hippocampus. As mentioned before, the EVF2 lncRNA is essential for the development of GABAergic neurons, as it regulates their gene expression in the developing brain. Accordingly, EVF2 silencing has been shown to cause abnormal formation of GABAergic circuits in the hippocampus and dentate gyrus, hence leading to abnormal synaptic activity in mice [94]. Translational control of local protein synthesis represents another crucial process underlying synaptic plasticity. As previously noted, the BC1/BC200 lncRNA is transported to synapses, where it represses local translation. Importantly, BC1/BC200 expression is dynamically upregulated in synapses in response to increased neuronal activity [106], thus modifying the protein composition of synapses with a negative feedback mechanism. Interestingly, knockout of BC1/BC200 in mice produces neuronal hyperexcitability and behavioral phenotypes, including increased anxiety and exploratory behavior defects [102,103,104]. Altogether, these data indicate that the translational control exerted by BC1/BC200 is essential for neuronal plasticity and its loss causes abnormal neuronal activity and behavioral defects. Alterations in the subunit stoichiometry of ion channels may also influence neuronal excitability and plasticity by affecting action potential thresholds. An example is provided by potassium voltage-gated channel subfamily A member 2 (KCNA2), a major potassium channel subunit, whose expression is regulated by the overlapping antisense RNA KCNA2-AS in response to peripheral nerve injury and in neuropathic pain [107]. The expression of KCNA2-AS is strongly induced by myeloid zinc finger 1 (MZF1) transcription factor in dorsal root ganglia (DRG) rat neurons upon nerve injury and its overexpression leads to a selective reduction of KCNA2 levels. In turn, this reduces the total voltage-gated potassium current and increase neuronal excitability, causing mechanical and pain hypersensitivity. These results show that KCNA2-AS is able to respond to peripheral nerve damage by altering synaptic plasticity. Interestingly, downregulation of another well characterized lncRNA, nuclear paraspeckle assembly transcript 1 (NEAT1), leads to changes in the expression of several genes involved in ion channel function following neuronal activation, as discussed below [108]. Another gene negatively regulated by an overlapping antisense RNA is *BDNF*, whose expression is repressed by BDNF-AS, as mentioned before. BDNF controls synaptic maintenance or elimination, depending on activity levels. Interestingly, expression of BDNF-AS is activity-dependent [83,99] and it has been hypothesized that it may control synaptic turnover and neuronal plasticity by coupling neuronal activity to BDNF expression [88]. Other lncRNAs have been proposed to couple neuronal activity and synaptic plasticity to alternative splicing of specific genes. For example, GOMAFU has recently been shown to modulate alternative splicing of DISC1 and ERBB4 [84], as already mentioned. Since deletion of *Erbb4* in mice enhances LTP in the hippocampus, GOMAFU-regulated splice isoforms of this gene may affect normal synaptic plasticity. Also, knock-down and overexpression of MALAT1, which is able to recruit different splicing factors, can reduce and increase synaptic density, respectively [105].

## 4. LncRNAs and Neuropsychiatric Disorders: The Three Musketeers in the Synapse—GOMAFU, NEAT1, and MALAT1

**Figure 1:**
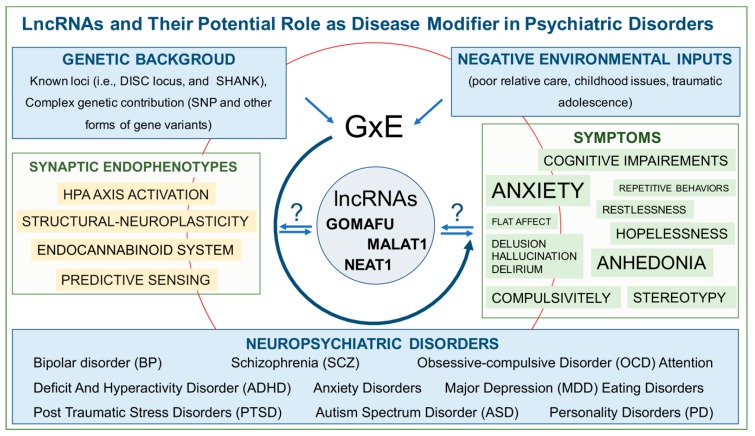
LncRNAs and their potential roles as disease modifiers in psychiatric disorders. Genome per environment (GxE)-related etiologic causes of mental illness entail molecular dysfunctions (endophenotypes) that impact synaptic function and cause a cluster of recurrent symptoms within different pathologic forms. The three better studied lncRNA, namely GOMAFU, NEAT1, and MALAT1, are implicated as disease-relevant factors. They could participate in pathologic manifestations directly, playing a role in pathophysiology, or could act as disease modifiers whose targeted modulation may represent a potential new strategy to ameliorate symptomatology. LncRNAs (Long non-coding RNAs); DISC locus (Disrupted in Schizophrenia locus); SNP (Single Nucleotide Polymorphism); HPA axis (Hypothalamic-Pituitary-Adrenal axis).

Some studies have already shown thorough modifications of lncRNAs expression within the brain of neuropsychiatric patients [84,109]. These studies outlined that lncRNAs are profoundly altered whenever normal neurophysiological processes are modified, suggesting that in conditions of altered neuronal physiology, lncRNA modulation could represent both a benign attempt to functionally adapt to an unbalanced condition or contribute to pathology development (Figure 1). In the second eventuality, multiple genetic factors alone or in combination with environmental challenges that modify the biology of lncRNAs in terms of expression or cellular localization could involve this class of epigenetic regulators as downstream pathologic effectors.

There is evidence that clearly implicates lncRNAs as part of the mechanisms involved in neuronal activity responses. In particular, their covariation of expression with the immediate early genes (IEGs) suggests an important involvement in neuroplasticity through synaptic tuning, consequently displaying the ability to impinge on circuitry excitability. The fact that lncRNAs are thoroughly modified in the post-mortem brain from neuropsychiatric patients is consistent with the widely accepted—albeit still to be fully demonstrated—neuroplasticity hypothesis of neuropsychiatric disorders. Within this frame, we now discuss several examples of experience-driven lncRNA modulation and the roles of lncRNAs as possible behavioral modifiers through the regulation of synaptic physiology.

### 4.1. GOMAFU—Anxiety Modifying lncRNA

GOMAFU (MIAT) represents a unique and remarkable example of well-investigated brain-relevant lncRNA. Indeed, GOMAFU’s role in neuropsychiatric disorders was proven through multidisciplinary approaches coupling clinical investigations—with reported deregulation in human post-mortem cortices of SCZ patient—to convincing basic science indicating activity-dependent behavior of this lncRNA [84,110]. GOMAFU levels decrease in response to neuronal activation, both in vitro and in vivo. Indeed, KCl-induced depolarization of mouse primary cultured neurons and human iPSC-derived neurons, together with induction of neuroplasticity-related programs of gene expression, also entails transient GOMAFU downregulation. Many brain areas involved in stress response, including the hippocampus, medial prefrontal cortex, and the amygdala, undergo rapid circuitry activation when a stressful paradigm is experienced. A widely used stressful learning paradigm in rodents involves fear conditioning. In this trial, a conditioned stimulus (CS, which can be the context cage, a sound, or a light) is followed by the unconditioned stimulus (US, the foot shock). This paradigm, entailing a training phase of repeated CS+US, has been widely used to assess associative memory consolidation between US and CS. However, as it represents a highly traumatic experience that also increases anxiety, it can be employed to monitor the molecular basis of stress response and anxiety arousal. Interestingly, fear conditioning (CS+US training), but not CS alone, significantly decreases GOMAFU expression in the mouse medial prefrontal cortex. Notably, from a behavioral point of view, GOMAFU knock-down, obtained by antisense oligonucleotides stereotaxically infused into the mPFC, is able to modify the basal level of anxiety [110]. It has been proposed that GOMAFU negatively controls a program of gene expression via a cis-mediated mechanism, including the *Crybb1* gene, which is involved in sustaining anxiety. The proposed epigenetic mechanism entails GOMAFU-mediated recruitment of polycomb repressive complex 1 (PRC1), and in particular its component BMI1, in the *Crybb1* promoter. In this regard, transient GOMAFU downregulation in response to anxiety-inducing trials such as fear conditioning could allow timely expression of genes whose transcription promotes increased vigilance and adaptive fear manifestation. However, this pro-anxiety transcriptional program should be limited within the duration of threatening or dangerous experiences. In this regard, SCZ patients, experiencing excessive, decontextualized expression of fear and anxiety underlying delusional disorders of the persecutory type, also show GOMAFU-stable downregulation [84]. GOMAFU knock-out mice were recently generated [111]. Interestingly, they did not display obvious morphological or physical differences to wild-type littermates, suggesting that this kind of mutation does not impact core biological processes. An interesting peculiarity of GOMAFU knock-out mice, however, is represented by hyperlocomotion behavior, which is not associated with emotional or affective issues but still represents a feature that has to be taken into consideration, given that rodent pharmacological models of SCZ display the same phenotype.

At this point, (i) GOMAFU stress-dependent downregulation, (ii) its facilitating role in pro-anxiety transcriptional program, and (iii) its stably reduced expression in SCZ post-mortem cortices strongly support the hypothesis of an important pathogenic role of GOMAFU in SCZ.

In further support of GOMAFU’s role in SCZ pathogenesis, considering GOMAFU’s function as a splicing regulator, overexpression of GOMAFU in human iPSC-derived neurons leads to significantly decreased levels of disrupted on schizophrenia 1 (DISC1), one of the most important loci associated with the disorder, along with DISC1 splicing isoforms Esv1, d3, and d7/8. Conversely, GOMAFU knock-down only increases DISC1 expression but not expression of its unspliced transcripts, another characteristic that is almost perfectly recapitulated in the SCZ post-mortem brain [84].

### 4.2. NEAT1—Excitability Modifying lncRNA

Nuclear-enriched abundant transcript 1 (NEAT1) belongs to a family of lncRNAs displaying considerable enrichment in the mammalian brain [112]. NEAT1 represents a critical scaffolding component and structural determinant of paraspeckles [113], subnuclear structures that are involved in many cellular functions, including splicing and transcriptional modulation through the modification of the chromatin structure [112]. Similarly to GOMAFU, NEAT1 also displays an aberrant pattern of expression in SCZ-patient-derived post-mortem specimens, in particular at the cortical level, where NEAT1 is significantly decreased in 14 subareas of the cerebral cortex [109], and also in the hippocampus, caudate, and putamen. Interestingly, the decrease of NEAT1 is independent on antipsychotic medications, as patients who suspended the therapy from 4 weeks to 7 years prior to death did not show significant differences of NEAT1 downregulation compared to treated patients. Notably, knock-out mice have been generated lacking the NEAT1 gene [114]. These mice are viable and fertile, and they do not display prominent histopathological modifications, except that they completely lack paraspeckles, suggesting that at least in resting conditions these subnuclear structures and their essential component NEAT1 are dispensable [114]. It was reported that NEAT1 plays an important role in unfolded protein response, as its levels significantly increased after a specific cellular challenge, i.e., proteasomal inhibition [113]. A possible interpretation is that NEAT1^KO^ mice could display specific vulnerability to cellular or environmental stress [114], a hypothesis that has not yet been explored.

Interesting suggestions of NEAT1’s implications in neurophysiology have been recently observed in in vitro and in vivo studies. NEAT1 follows GOMAFU’s trend in the SCZ post-mortem brain. Similarly to GOMAFU, NEAT1 expression is sensitive to neuronal activation. Indeed, KCl-induced depolarization of human induced pluripotent stem cell (iPSC)-derived neurons rapidly and transiently elicits NEAT1 downregulation. RNA immunoprecipitation technique showed interaction of NEAT1 with KCNAB2 and KCNIP1 in SH-SY5Y cells. These modulatory proteins aim to decrease neuronal excitability, buffering excessive synapse depolarization, a behavior that is also engaged upon KCl-induced neuronal activation [108]. Thus, a picture emerges in which an activity-dependent decrease of NEAT1 levels could be involved in free protein interactors, allowing nuclear-cytoplasmic shuttling of KCNAB2 and homeostatic enhancement of potassium channel activity, aiding the shut-off of synapses [108]. Importantly, NEAT1 downregulation is also acutely promoted in rats with pharmacological epilepsy with two different chemo-convulsant compounds (pilocarpine and kainic acid). These two models of temporal lobe epilepsy act by increasing glutamatergic neurotransmission in temporal brain areas. Interestingly, upon chronic epileptogenic stimulation, NEAT1 levels become unresponsive to neuron hyperexcitation, increasing the risk of seizure activity [108]. From these data, the authors draw a clear implication of acute downregulation of NEAT1 levels in hyperexcitability protection, which is lost upon reiterated engagement of such a process and is highly consistent with increased NEAT1 levels in the brains of patients affected by intractable seizures [108]. If we take into consideration the seemingly counterintuitive observation that antisense oligonucleotide (ASO)-mediated NEAT1 downregulation increases neuronal excitability in vitro [108], possibly through scaling-up of homeostatic synapses, we could depict a possible role of NEAT1 downregulation in the homeostatic synaptic scaling-up of SCZ patients with increased cortical circuitry over-excitability. Alternatively, it is also possible that in a complex in vivo circuital context such as the human cortex, NEAT1 downregulation may be uncoupled from mechanisms of homeostatic plasticity; in this second scenario, NEAT1 deregulation could be instrumental in decreasing cortical excitability. Interestingly, both these scenarios are compatible with disrupted predictive sensing, a core symptom of neuropsychiatric disorders [11,12,36].

### 4.3. MALAT1—Neuroplasticity Modifying lncRNA

Until now we have described literature data involving coupling molecular lncRNA-related modifications—which are associated or even causally linked to interesting endophenotypes in terms of neuronal excitability and behavior—with clinical data showing that the same lncRNAs, namely GOMAFU and NEAT1, undergo thorough deregulation in the schizophrenic brain. Notably, another lncRNA, MALAT1, which displays considerable expression in neurons, can be linked thanks to rodent models of SCZ to the set of molecular alterations contributing to disease onset and progression. The SCZ-like phenotype can be effectively induced by chronically inhibiting the ionotropic glutamate receptor NMDAR with open-pore blockers, including MK-801 and ketamine. Interestingly, such models also work in humans and non-human primates, which substantially enhances their validity. Indeed, chronic inhibition of the NMDAR reproduces core SCZ-like traits (such as hallucinations and negative symptoms, which are clearly not easily measurable in rodents), cognitive deficits [115], and well-known SCZ endophenotypes in terms of gamma oscillation disruption, both in rodents and primates [116]. In rodents, a behavioral marker of SCZ-like acquired symptomatology is increased locomotor activity, which is also induced by methamphetamine and amphetamine activation of dopaminergic receptors, similarly impacting gamma oscillation and representing similar pharmacological models of SCZ. Remarkably, both chronical MK-801-mediated inhibition of NMDAR and methamphetamine administration, along with inducing core SCZ symptomatology in mice, also cause lncRNA modification that is highly reminiscent of modifications observed in the SCZ human brain [115,116,117]. Indeed, these two treatments induce significant downregulation of GOMAFU, a clear trend toward decreased NEAT1, and a strong decrease in MALAT1 [117]. We previously focused our attention on neuronal activity-dependent covariation of widely known neuroplasticity-related genes (in particular the IEGs) and lncRNAs. In this regard, MALAT1 represents an eminent example of activity-modulated lncRNA, whose transcriptional induction mechanism interestingly retraces those of the IEGs. These genes respond to neuronal activation with a peculiar fast-track-like epigenetic mechanism recently described by Li Huei Tsai’s group [118], which is aimed at instant transactivation that perfectly matches stimuli perception. It has been referred to as the double-strand brake (DSB) of gene promoters. This mechanism utilizes the DNA double-strand brake as a way of speeding up RNA pol2 processivity, resolving the topological constraints of double-helix DNA. MALAT1 follows the same DSB-accelerated transactivation process. These data suggest that the peculiar fast kinetic nature of MALAT1 transcriptional response in neurons is related to a stimuli-processing role in neuroplasticity. Considering that lncRNAs are ready-to-use molecules, neurophysiology changes upon experience might, at least theoretically, be primed by MALAT1.

Notably, all three lncRNAs belong to the class of lincRNAs (defined as lncRNAs which do not overlap with coding regions), reasonably having a multitarget “trans” effect, as witnessed by their pleiotropic activity.

## 5. Concluding Remarks

In conclusion, the complex nature of modified lncRNA transcriptomes must include relevant genes: (i) with pathogenic implications, participating to disease onset and progression; (ii) with homeostatic properties, such as those lncRNAs whose variation represents an attempt to counteract pathophysiology; or (iii) neutral with respect to the disease. We strongly believe that the role of lncRNAs in neuroplasticity modulation endows many post-mortem transcriptional studies with a novel role of interpretation involving at least those lncRNAs species that undergo activity-induced modulation.

The role of lncRNAs in psychiatric disorders has just started to be investigated, and we can now only outline their functional implications in pathology onset and progression. For instance, the observations made in schizophrenia research could be extended to other neuropsychiatric disorders in the context of the ever-growing idea of a continuum among these pathologies [9].

In general, the numbers of protein coding genes, as well as their functions, are comparable from lower to higher eukaryotes. On the contrary, lncRNAs are highly specific to primate lineages and the majority of them are restricted to neurons. This context sheds light on the role of lncRNAs in driving the evolution of brain complexity. It seems that neuropsychiatric disorders are peculiar to mammals, specifically those species enriched in lncRNAs. As all multicellular organisms have nervous systems and neurons, the further layer of regulation in lncRNAs fuels an extraordinary emergent nature that is not instrumental to the biological processes but is involved in finely shaping the highest mental functions. Nonetheless, the higher the inherent complexity, the more potential issues. Luckily, in circumstances of fine circuity disruption (or probably by their virtue), humans are able to create extraordinary art and make amazing technological and scientific discoveries. In other words, as Randolph Nesse recently argued, “there must be a good reason for bad feelings” [119].

## Figures and Tables

**Table 1:** Comparison between the number of coding and non-coding lncRNA genes across six different species, along with their evolution. Note that while the number of coding genes does not increase along with species complexity, the number of lncRNAs increases considerably. Sources: ^1^ GENCODE (https://www.gencodegenes.org/); ^2^ Ensembl Genome Browser (https://www.ensembl.org/); ^3^ FlyBase (https://flybase.org/); ^4^ Saccharomyces Genome Database (SGD) (https://www.yeastgenome.org/). LncRNA, long non-coding RNA.

An Emerging Role of lncRNAs in Driving Evolution		
Species	Number of Protein-Coding Genes	Number of lncRNA Genes
*Homo sapiens*	19.957 ^(1)^–20.449 ^(2)^	16.900 ^(2)^–17.952 ^(1)^
*Mus musculus*	21.856 ^(1)^–22.515 ^(2)^	9.981 ^(2)^–13.197 ^(1)^
*Danio rerio*	25.592 ^(2)^	3.278 ^(2)^
*D. melanogaster*	13.968 ^(3)^	2.539 ^(3)^
*C. elegans*	20.191 ^(2)^	276 ^(2)^
*S. cerevisiae*	6.604 ^(4)^	18 ^(4)^
